# Histochemical Properties of the Vomeronasal System in Hokkaido Sika Deer (*Cervus nippon yesoensis*)

**DOI:** 10.3390/ani15233475

**Published:** 2025-12-02

**Authors:** Daisuke Kondoh, Toshiki Arimura, Mimi Arakaki, Yukiko Otake, Teruhiro Kanagawa, Jumpei Tomiyasu

**Affiliations:** 1 Department of Veterinary Medicine, Obihiro University of Agriculture and Veterinary Medicine, Inada-cho Nishi 2-11, Obihiro 080-8555, Japanj.tomiyasu@obihiro.ac.jp (J.T.); 2 Shiretoko Nature Foundation, Shari 099-4356, Japan; t_kanagawa@shiretoko.or.jp

**Keywords:** artiodactyls, ecology, olfaction, pheromone, reproductive behavior, ruminants, sensory system

## Abstract

The vomeronasal system (VNS) is directly linked to the various behavior and ecology of all animal species, and understanding it might help to prevent deer damage. We therefore histochemically analyzed the accessory olfactory bulb (AOB) and the vomeronasal organ (VNO) that, respectively, function as a primary integrative center and a peripheral receptor organ, in Hokkaido sika deer (*Cervus nippon* ssp. *yesoensis*). The AOB consisted of the vomeronasal nerve, glomerular, plexiform, and granule cell layers. We found that G protein α subunit i2 (Gαi2) and o (Gαo) that are, respectively, coupled with vomeronasal receptor type 1 and 2 families were strongly and weakly expressed in the glomerular layer, respectively. These properties of the AOB of sika deer were similar to those of other artiodactyl species, including wapiti. We then explored the sika deer VNO using 21 lectins that bind to each glycan structure. Although various cell types in the VNO had unique lectin binding profiles, all 21 lectins bound to the free border of the sensory epithelium, suggesting that various glycoconjugates are involved in pheromone detection in sika deer via the VNO. Furthermore, the reactivity of some lectins in the sensory epithelium and vomeronasal gland differed from those of roe deer and wapiti. Our findings suggest that the composition of glycoconjugates in the VNO differs among deer species.

## Simple Summary

The vomeronasal system (VNS) is directly linked to various behaviors, including reproduction and ecology, of deer species, and understanding it might help to prevent deer damage. Here, we analyzed the accessory olfactory bulb (AOB) and the vomeronasal organ (VNO) of Hokkaido sika deer (*Cervus nippon* ssp. *yesoensis*) by immunohistochemistry, and the VNO was also analyzed by lectin histochemistry. The properties of the AOB of sika deer were similar to those of roe deer and wapiti. On the other hand, lectin binding profile of the VNO in sika deer differed from that in roe deer or wapiti, indicating that even among closely related species, the composition of glycoconjugates in the region where vomeronasal receptors are expressed differs. This is an example of why findings should not be easily extrapolated, even to species belonging to the same family.

## 1. Introduction

Ruminant deer belong to the Cervidae family, which currently composes ~55 species. Deer populations have recently increased exponentially around the world, which has impacted ecosystems and contributed to the transmission of various diseases to humans and other animals [1]. Therefore, measures are needed from various perspectives, including breeding patterns and ecology in the wild.

The sensory vomeronasal system (VNS) in mammals detects the species-specific substances such as pheromones and kairomones. Estrus-specific compounds in urine and other fluids function in ruminant livestock as pheromones and help to identify reproductive status [2]. These pheromones are uptaken via flehmen, a behavior that has been thoroughly investigated in cattle [3], goats [4], and sheep [5]. Flehmen behavior occurs in many wild ruminant species such as giraffes [6], antelopes [7], and deer [8,9]. Thus, the VNS is common in ruminants, and understanding this system in various species is important for their management.

The primary integrative center of the VNS in the brain is the accessory olfactory bulb (AOB), which is a specialized part of the olfactory bulb found in ruminant white-tailed deer (*Odocoileus virginianus*) [10], Siberian roe deer (*Capreolus pygargus*) [11], and wapiti (*Cervus canadensis*) [12]. The AOBs of roe deer [11] and wapiti [12] histologically consist of vomeronasal nerve (VNL), glomerular (GL), plexiform (PL), and granule cell (GCL) layers. The PL contains mitral/tufted cells that are output neurons of the AOB. The types 1 (V1R) and 2 (V2R) vomeronasal receptor families function in mammals by associating with the G protein α subunits i2 (Gαi2) and -o (Gαo), respectively [13], and they bind different ligands [14,15]. The VNS expresses both types in some species whereas others express only V1Rs. When both types are expressed in the VNS, the anterior and posterior halves of the AOB are immunohistochemically positive for Gαi2 and Gαo (segregated type), respectively [16,17,18,19,20,21,22,23,24,25], whereas the entire AOB is Gαi2 positive when only V1Rs are expressed (uniform type) [12,26,27,28,29].

The peripheral receptor organ of the VNS is the vomeronasal organ (VNO), which consists of a pair of tubular structures containing sensory cells in the anterior part of the nose. The VNO is morphologically sophisticated in ruminants such as cattle [30,31,32], goats [26,33], sheep [27,34,35], giraffes [36], antelopes [37], and the deer species, roe [11], moose (*Alces alces*) [38], reindeer (*Rangifer tarandus*) [9], wapiti [12], and sika (*Cervus nippon*) [39]. Lectin proteins bind specific terminal carbohydrates on glycans [40], and are extensively used to distinguish cell types [41]. Because the VNOs of roe deer [42] and wapiti [12] have been histochemically examined using 21 and 10 lectins, respectively, screening sika deer VNOs using lectins can help to clarify similarities and differences among deer species.

Here, we applied an immunohistochemical approach to investigate the AOB and the VNO of the Hokkaido sika deer (*Cervus nippon* ssp. *yesoensis*). We also explored the VNO using lectin histochemistry to verify the similarity and diversity of VNS among various deer species.

## 2. Materials and Methods

### 2.1. Animals

We analyzed heads from two adult female (SD-2 and SD-3) and two adult male (SD-1 and SD-4) Hokkaido sika deer that had been harvested during January 2025 under a government commission for population control purposes. The nasal regions comprising the VNO and the brain, including the olfactory bulbs, were removed within three hours after death and preserved in 10% formalin. The Animal Care and Use Committee of Obihiro University of Agriculture and Veterinary Medicine was notified of the experimental protocol (notification numbers: 24-9), and the study proceeded according to our Institutional Regulations on the Management and Operation of Animal Experiments.

### 2.2. Antibodies and Lectins

We obtained the primary antibodies as follows: 1:100-diluted sc-67219 rabbit anti-olfactory marker protein (OMP) (human origin), 1:50-diluted sc-13534 mouse anti-Gαi2 (human origin), and 1:100-diluted sc-387 rabbit anti-Gαo (rat origin) (Santa Cruz Biotech., Dallas, TX, USA). The amino acid sequences of these three molecules have been identified in the wapiti (*Cervus canadensis*), a species of the same genus as sika deer. The amino acid sequences of OMP and Gαi2 in human and wapiti were 85.9% and 99.4% identical, respectively, and the sequence of Gαo in rat (*Rattus norvegicus*) and wapiti were 92.4% identical.

The secondary antibodies were biotinylated goat polyclonal antibodies against mouse IgG (1:200; BA-9200) or rabbit IgG (1:200; BA-1000) (Vector Laboratories Inc., Burlingame, CA, USA).

The histochemical characteristics of the VNO were evaluated using 21 biotinylated lectins (Table 1) including lectin screening kits I–III (Vector Laboratories Inc.).

### 2.3. Histological Procedures

Olfactory bulbs were fixed in 10% formalin for one week, dehydrated, and embedded in paraffin. The nasal region with the VNO was also fixed in 10% formalin for one week, decalcified in Prank-Rychlo solution (Muto Pure Chemicals Co., Ltd., Tokyo, Japan) for 24 h, then embedded in paraffin. The AOBs were sliced sagittally into 5 μm thick sections for hematoxylin and eosin (HE) staining or immunohistochemistry. The VNOs were sliced frontally into 5 μm thick sections for HE, periodic acid-Schiff (PAS), or Alcian blue (pH 2.5, AB) stainings, or for lectin histochemistry.

### 2.4. Immunohistochemical Protocol

Some sections including the AOB were processed for anti-OMP, -Gαi2, and -Gαo immunostainings as described in [28]. Briefly, deparaffinized sections were microwaved in Tris-EDTA buffer (pH 9.0) and then incubated with 0.3% H_2_O_2_ in methanol followed by 3% normal goat serum. The sections were incubated with primary antibodies overnight at 4 °C, then with biotinylated secondary antibody for 1 h. The sections were reacted with the avidin–biotin complex reagent (PK-6100; Vector Laboratories Inc.), then stained using 0.02% 3,3′-diaminobenzidine tetrahydrochloride in Tris-HCl buffer containing 0.006% H_2_O_2_ (DAB solution). Primary antibodies were replaced by normal goat serum in control sections.

### 2.5. Lectin Histochemistry

We processed VNO sections for lectin histochemistry as described [43]. Briefly, deparaffinized sections were incubated with 0.3% H_2_O_2_ in methanol, followed by 1% bovine serum albumin. The sections were incubated with various concentrations of biotinylated lectins (Table 1) in phosphate-buffered saline-0.5% Triton X at 4 °C overnight, immersed in avidin–biotin–peroxidase complex reagent, then visualized using DAB solution for ~10 min. Lectins were replaced by 1% bovine serum albumin in control sections.

### 2.6. Scoring of Staining Intensity

Staining intensity was evaluated as negative, weak, moderately, or strongly positive based on the correlation between the images and the scoring shown in the study on the roe deer VNO (Figures 4 and 5 and Tables 2 and 3 in [42]). Nonspecific weak staining that was indistinguishable from the background was defined as negative.

## 3. Results

### 3.1. Histological and Immunohistochemical Features of AOB

**Figure 1:**
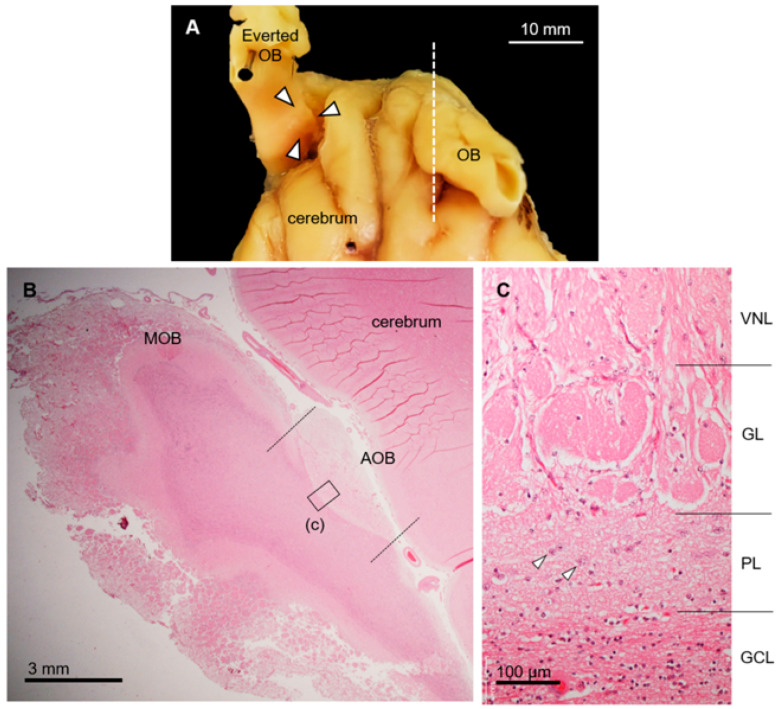
Morphological and histological features of accessory olfactory bulb (AOB) in sika deer. (**A**) Location of AOB (arrowheads) after everting olfactory bulb (OB). Dorsal view of anterior part of brain. (**B**) Histological section corresponds to dashed line in panel (**A**). Hematoxylin–eosin staining. Left, anterior; upper, dorsal. MOB, main olfactory bulb. (**C**) Higher magnification of image corresponding to box (c) in panel (**B**). Accessory olfactory bulb consists of vomeronasal nerve (VNL), glomerular (GL), plexiform (PL), and granule cell (GCL) layers. Arrowheads, mitral/tufted cells. This section is from a female (SD-2), and there is no significant difference between females and males (Supplementary Figure S1).

**Figure 2:**
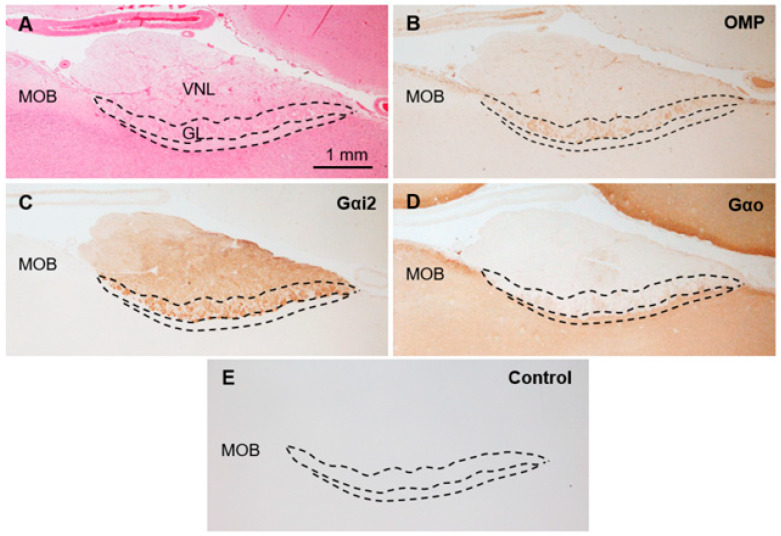
Immunohistochemical properties of sika deer AOB. (**A**) Hematoxylin–eosin staining. GL, glomerular layer; MOB, main olfactory bulb; VNL, vomeronasal nerve layer. (**B**–**D**) Immunostaining against anti-olfactory marker protein (OMP) (**B**), anti-G protein α subunit i2 (Gαi2) (**C**), and anti-G protein α subunit o (Gαo) (**D**). (**E**) Negative control section. These sections are from a female (SD-2), and there is no significant difference between females and males (Supplementary Figure S1).

The sika deer AOB was a large structure located at the medial side of the olfactory bulb and posterodorsal to the main olfactory bulb (Figure 1A,B) consisting of the VNL, GL, PL, and GCL (Figure 1C). Mitral/tufted cells were found in the PL. Anti-OMP reacted with the glomerular layer, and anti-Gαi2 reacted strongly with the VNL and GL (Figure 2A–C). Anti-Gαo reacted moderately with the PL and GCL, weakly with the GL, and not at all with the VNL (Figure 2D). Glomeruli in the main olfactory bulb were positive for anti-OMP and -Gαo, but negative for anti-Gαi2 (Figure 2). There was no significant difference in the features of the AOB between females and males (Supplementary Figure S1).

### 3.2. Histological Features of the Deer VNO

**Figure 3:**
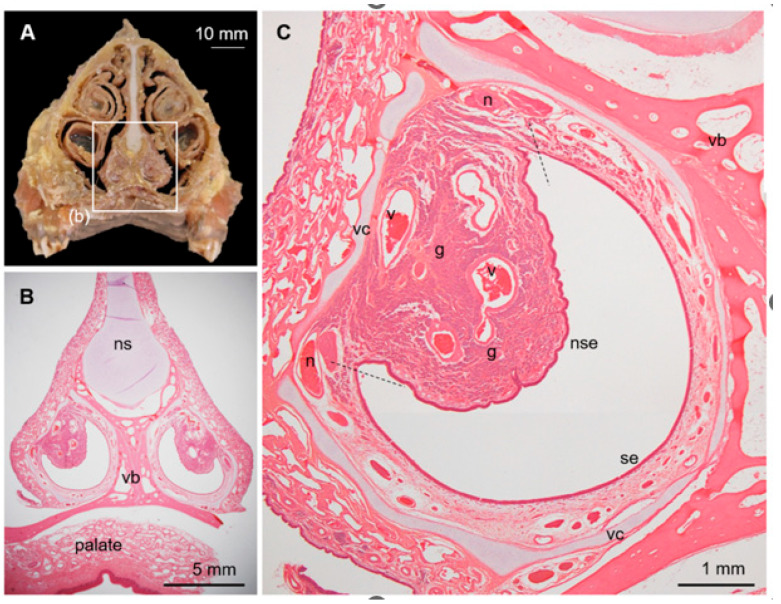
Vomeronasal organ (VNO) in sika deer. (**A**) Cross-section of nasal structure of sika deer. (**B**) Histological section corresponding to box (b) in panel (**A**). Hematoxylin–eosin staining. ns, nasal septum; vb, vomer bone. (**C**) Histological components of VNO. Hematoxylin–eosin staining. Dashed lines indicate border between sensory (se) and non-sensory (nse) epithelia. g, vomeronasal glands; n, axon bundles (vomeronasal nerve); v, venous sinus; vc, vomeronasal cartilage. This section is from a female (SD-2), and sections from all individuals analyzed are shown in Supplementary Figure S2.

The deer VNO comprised a pair of large tubular structures with a diameter of ~5 mm at the base of the nasal septum (Figure 3A,B). The vomeronasal cartilage surrounded almost all the entire soft tissues, and the VNO lumen was wide and crescent-shaped (Figure 3C). The lateral and medial sides of the lumen were covered by non-sensory and sensory epithelium, respectively. These two epithelia were bounded on the ventral and dorsal sides, where the gland ducts open. The lateral soft tissues bulged luminally and contained many gland acini and some large veins. Medial axon bundles converged near the border between the sensory and non-sensory epithelia to form thick vomeronasal nerves.

**Figure 4:**
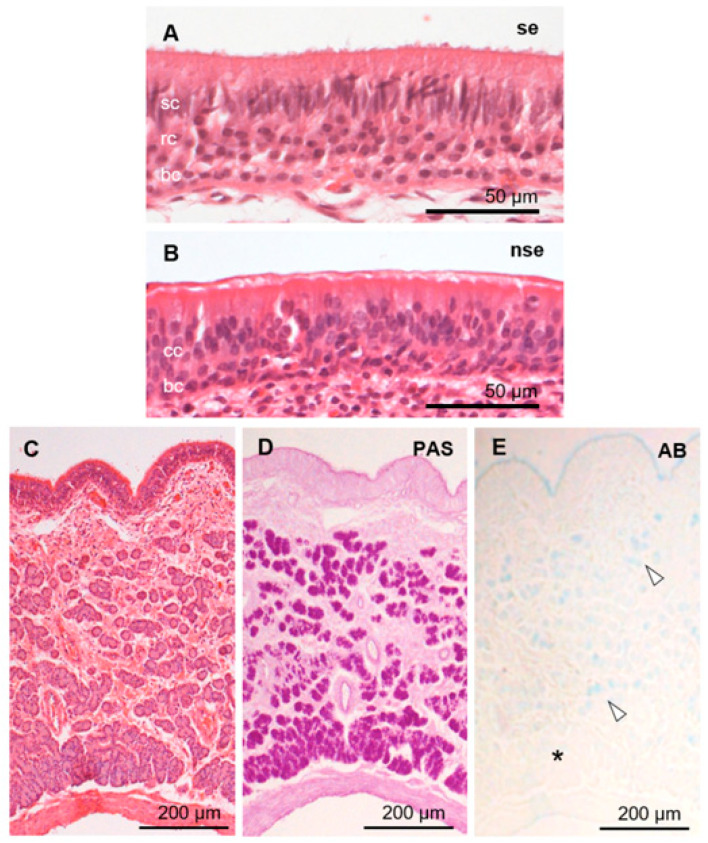
Histological features of sika deer VNO. Sensory (se; **A**) and non-sensory (nse; **B**) epithelia, and vomeronasal glands (**C**,**D**). Hematoxylin–eosin (**A**–**C**), periodic acid-Schiff (PAS; **D**), and Alcian blue pH 2.5 (AB; **E**) stainings. Arrowheads and asterisk in panel (**E**) indicate AB-positive and -negative acini, respectively. bc, basal cells; cc, ciliated columnar cells; rc, receptor cells; sc, supporting cells. These sections are from a female (SD-2), and sections from all individuals analyzed are shown in Supplementary Figure S3.

**Figure 5:**
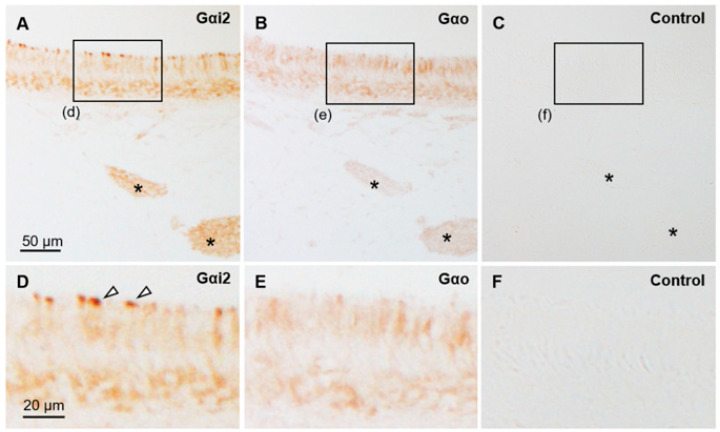
Immunohistochemical properties of sensory epithelium in sika deer VNO. (**A**–**C**) Immunostaining against anti-Gαi2 (**A**) and anti-Gαo (**B**) and negative control (**C**). Asterisks indicate axon bundles, and boxes (d–f) correspond to an area showing in panels (**D**–**F**), respectively. (**D**–**F**) High-magnified images of sensory epithelium. Arrowheads indicate Gαi2-positive dendritic knobs. The control image shown in (**C**) is very similar to the lectin staining control image in Figure 6. These sections are from a female (SD-3).

The sensory epithelium of the VNO was pseudostratified and consisted of supporting, receptor, and basal cells (Figure 4A). Supporting cells had elongated nuclei arranged in one or two rows on the luminal side. Receptor cells with round nuclei were identified in the middle of the epithelium in one to three cell layers. Basal cells also had round nuclei and were arranged in a single row on the basal side. Dendritic knobs of the receptor cells reacted strongly with anti-Gαi2 (Figure 5), similar to that in a previous report [39]. The cell body of receptor cells and axonal bundles below the epithelium reacted moderately and weakly with anti-Gαi2 and anti-Gαo, respectively (Figure 5). The non-sensory epithelium of the VNO comprised columnar cells with cilia and basal cells (Figure 4B). About half of the PAS-positive mucous vomeronasal glands were AB-positive, and the other half were AB-negative (Figure 4C–E). There was no significant difference in the VNO characteristics between females and males (Supplementary Figures S2 and S3).

### 3.3. Lectin Bindings in the Sika Deer VNO

**Figure 6:**
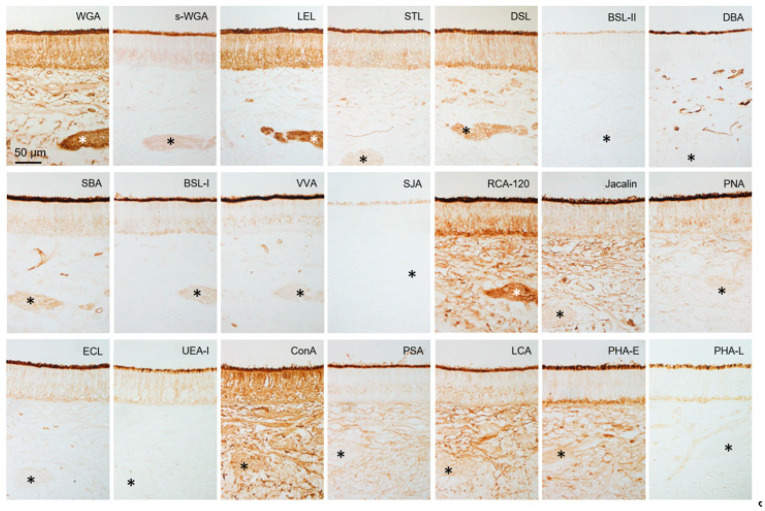
Lectin binding in sensory epithelium of sika deer VNO. Reactivity to lectins (Table 1), which is summarized in Table 2. * Axon bundles. These sections are from a male (SD-4), and there is no significant difference between females and males (Supplementary Figure S4).

**Figure 7:**
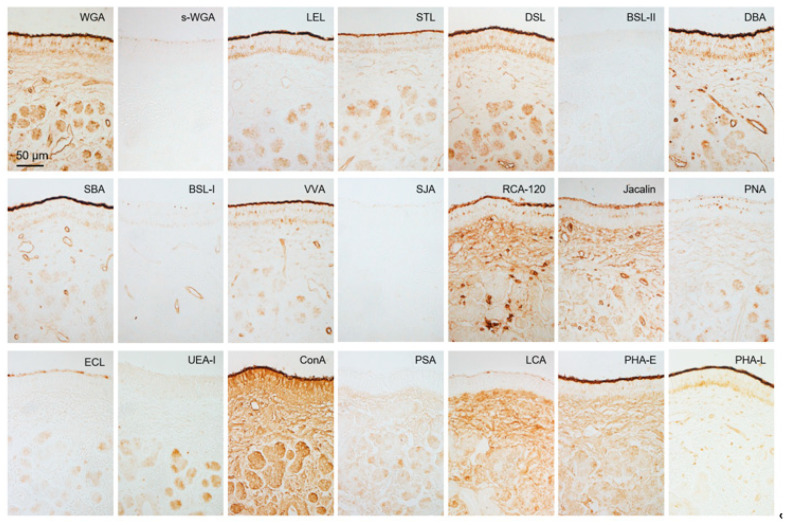
Lectin bindings in non-sensory epithelium and vomeronasal glands. Reactivity to 21 lectins (Table 1). This is summarized in Table 2. These sections are from a male (SD-4), and there is no significant difference between females and males (Supplementary Figure S5).

Figure 6 and Figure 7 show that the free border of the sensory epithelium was positive for all 21 lectins tested, while the cell types of sensory and non-sensory epithelia had different lectin binding profiles (Table 2). The four sika deer samples similarly reacted to the 21 lectins in the VNO (Supplementary Figures S4 and S5).

## 4. Discussion

The present study revealed the histological characteristics of the AOB in sika deer. The four-layer structure of the AOB was similar to that of roe deer [11] and wapiti [12], and thus seems to be a common feature among deer species. The immunohistochemical findings in the AOB and the VNO indicated that sika deer have the uniform type of VNS with V1R-neurons, like other artiodactyl species [26,27,28,29] including wapiti [12]. The sensory epithelium of the VNO reacted weakly with anti-Gαo, but it was not localized to the dendritic knobs, and we conclude that it is not related to vomeronasal receptors. Because the cell bodies of the receptor cells in the sika deer VNO were completely negative in previous results using an anti-Gαo monoclonal antibody [39], the weak reaction with the anti-Gαo polyclonal antibody in the sensory epithelium and axon bundles in this study is thought to be a nonspecific false positive. We discuss that a Gαo-positive reaction in the AOB is also unrelated to V2R, for reasons detailed in the discussion of the previous report [29]. Briefly, Gαo is involved in development of the olfactory bulb [44], and thus immunoreaction for Gαo within the AOB has also been found in many mammals without V2R [19,28,29]. The V1Rs coupled with Gαi2 bind to low molecular weight odorants [45] including some steroid metabolites [46]. Thus, deer might use these substances as pheromones and kairomones.

OMP seems to play an important role in the olfactory system, such as odor transduction and refinement of the glomerular map, and its presence extends from the receptor cells to the glomeruli in the olfactory bulb [47]. Although the exact function of OMP remains poorly understood in the VNS, OMP is generally expressed in the VNL and GL of the mammalian AOB [18,27,28], which contains the axons of receptor cells. However, the GL of the sika deer AOB was positive for anti-OMP, whereas the VNL was negative. Interestingly, in the wapiti AOB, as in the sika deer AOB, OMP is positive in the GL and negative in the VNL [12], suggesting that the expression of OMP in the AOB of the Cervidae differs from that of other mammalian species. Further analysis of this deer-specific OMP expression, including its developmental and seasonal changes, is required.

The histological components of the sika deer VNO were similar to previous findings [39], but the vomeronasal glands were more developed, as we previously found [48]. We, and others [39], sampled deer during January, and could not differentiate males from females. Therefore, the difference in the vomeronasal glands is perhaps because of differences between subspecies such as *C. n. aplodontus* and *C. n. yesoensis*. The areas covered by sensory and non-sensory epithelium in the VNO of the sika deer coincided with those of the VNO of the roe deer [11] and wapiti [12]. An important difference between the VNO of sika and roe deer [11] is that goblet cells in the non-sensory epithelium are absent, as they are in wapiti [12]. The vomeronasal gland and goblet cells both contribute to forming the mucus layer for pheromone reception in roe deer, whereas only the vomeronasal gland contributes to such formation in sika deer and wapiti. This suggests diverse mechanisms of pheromone reception among deer species.

All 21 lectins tested herein bound to the free border of the sensory epithelium where pheromones bind to receptors as ligands in sika deer VNO. This suggests that various glycoconjugates are mechanistically involved in pheromone detection in sika deer. Furthermore, the different lectin binding profiles of various cell types indicated that these cells not only differ in terms of cell morphology and arrangement, but also in their expressed glycoconjugate profiles. Non-sensory epithelium showed different lectin binding profiles from sensory epithelium, suggesting that non-sensory epithelium express different glycan structures to achieve functions distinct from chemoreception, such as host defense, immunity, and ciliary movement.

Compared with lectin binding in the VNO of roe deer [42], the free borders of sensory epithelium in both species were positive for 21 types of lectins. This indicated that glycoconjugates herein are complex in both species, but the degree of similarity cannot be assessed from these results. Lectin reactivities of receptor cells were generally similar, except for that of *Phaseolus vulgaris* agglutinin-E (PHA-E). Therefore, glycoconjugates involved in conducting stimuli from the VNO to the AOB might be similar between these species. In contrast, the reactivity to six and five lectins in supporting and basal cells of the sensory epithelium differed between sika and roe deer, respectively. *Bandeiraea simplicifolia* lectin-II (BSL-II), *Vicia villosa* agglutinin (VVA), *Pisum sativum* agglutinin (PSA), PHA-E, and PHA-L reacted with supporting cells only in roe deer, and peanut agglutinin (PNA) reacted with those only in sika deer. *Dolichos biflorus* (DBA) and soybean (SBA) agglutinins, VVA, and PSA reacted with basal cells only in roe deer, and PNA reacted with those in sika deer. Therefore, glycoconjugates in supporting and basal cells differed between the sika and roe deer VNOs. Thus, the dynamics and functions of these cells might differ among deer species.

The reactivity to *Solanum tuberosum* lectin (STL) and DBA at the free border of the sensory epithelium of the sika deer VNO is stronger than that of wapiti [12], which belongs to the same genus as sika deer. The intensity of the ECL reaction in the vomeronasal gland was weaker in the VNO of sika deer compared with wapiti. These findings suggest that even among closely related species, the composition of glycoconjugates in the region where vomeronasal receptors are expressed differs, as do mucous components that dissolve pheromones.

## 5. Conclusions

Here, we histologically assessed the VNS of the sika deer in detail and explored the diversity within deer species. The VNS of deer species share some similarities, whereas secretion and lectin profiles differed, particularly in the VNO. This is an example of why findings should not be easily extrapolated, even to species belonging to the same family. As another example, it has recently been revealed that, even within the same Pinnipedia, sea lions have a well-developed VNS [49], while harbor seals have lost it [50]. Salazar and Sánchez-Quinteiro [51] have argued that it would be a major mistake to extrapolate VNS anatomical findings from one species to another, even between relatively close species, and this study strongly supports this notion.

## Figures and Tables

**Table 1:** Lectin concentrations and specificities.

Lectins	Abbreviation	Concentration (mg mL^−1^)	Rough Specificity
Wheat germ agglutinin	WGA	1.0 × 10^−2^	GlcNAc
Succinylated-wheat germ agglutinin	s-WGA	1.0 × 10^−2^	(GlcNAc)_n_
*Lycopersicon esculentum* lectin	LEL	2.0 × 10^−3^	(GlcNAc)_2–4_
*Solanum tuberosum* lectin	STL	1.0 × 10^−2^	(GlcNAc)_2–4_
*Datura stramonium* lectin	DSL	4.0 × 10^−3^	(GlcNAc)_2–4_
*Bandeiraea simplicifolia* lectin-II	BSL-II	5.0 × 10^−2^	α/βGlcNAc
*Dolichos biflorus* agglutinin	DBA	5.0 × 10^−2^	αGalNAc
Soybean agglutinin	SBA	1.0 × 10^−2^	α>βGalNAc
*Bandeiraea simplicifolia* lectin-I	BSL-I	5.0 × 10^−3^	αGal, αGalNAc
*Vicia villosa* agglutinin	VVA	1.0 × 10^−2^	GalNAc
*Sophora japonica* agglutinin	SJA	5.0 × 10^−2^	GalNAc
*Ricinus communis* agglutinin-I	RCA-120	2.0 × 10^−3^	Gal
Jacalin		5.0 × 10^−4^	Galβ3GalNAc
Peanut agglutinin	PNA	4.0 × 10^−3^	Galβ3GalNAc
*Erythrina cristagalli* lectin	ECL	2.0 × 10^−2^	Galβ4GlcNAc
*Ulex europaeus* agglutinin-I	UEA-I	5.0 × 10^−2^	αFuc
Concanavalin A	ConA	3.3 × 10^−3^	αMan, αGlc
*Pisum sativum* agglutinin	PSA	4.0 × 10^−3^	αMan, αGlc
*Lens culinaris* agglutinin	LCA	4.0 × 10^−3^	αMan, αGlc
*Phaseolus vulgaris* agglutinin-E	PHA-E	5.0 × 10^−3^	Galβ4GlcNAcβ2Manα6(GlcNAcβ4)(GlcNAcβ4Manα3)Manα4
*Phaseolus vulgaris* agglutinin-L	PHA-L	2.5 × 10^−3^	Galβ4GlcNAcβ6(GlcNAcβ2Manα3)Manα3

Fuc, fucose; Gal, galactose; GalNAc, N-acetylgalactosamine; Glc, glucose; GlcNAc, N-acetylglucosamine; Man, mannose.

**Table 2:** Reactivity of 21 lectins with vomeronasal organ of sika deer.

	Sensory Epithelium				Axon Bundles		Non-Sensory Epithelium			Gland Acini
	Free Border	Receptor Cells	Supporting Cells	Basal Cells			Free Border	Ciliated Cells	Basal Cells	
WGA	+++	+++	++	+++	+++		+++	++	++	++
s-WGA	++	+	−	−	+		++ (dots)	−	−	−
LEL	+++	+++	++	+++	+++		+++	+	++	++
STL	+++	++	+	+	+		+++	+	+	++
DSL	+++	++	+	+++	++		+++	++	++	++
BSL-II	+	−	−	−	−		−	−	−	−
DBA	+++	−	−	−	−		+++	++	++	++
SBA	+++	++	+	−	++		+++	+	+	+
BSL-I	+++	+	−	++	+		+++ (dots)	−	+	−
VVA	+++	++ (dots)	−	−	+		+++	+	+	++
SJA	+	−	−	−	−		+ (dots)	−	−	−
RCA-120	+++	+++	++	+++	+++		+++	+	−	+
Jacalin	+++	+	+	++	+		++	+	−	++
PNA	+++	++ (dots)	+	++	+		+++ (dots)	+	−	− or +++
ECL	+++	+	+	++	+		+++ (dots)	−	−	+
UEA-I	++	+	+	+	−		−	−	−	− or +++
ConA	+++	+++	+++	++	++		+++	+++	++	+++
PSA	+++	+ (dots)	−	−	−		−	−	−	+
LCA	+++	++ (dots)	+	+	+		+++ (dots)	−	−	+
PHA-E	+++	−	−	+++	+		+++	++	++	+
PHA-L	++	−	−	+	−		+++	+	++	+

−, negative; +, weakly; ++, moderately; +++, strongly positive.

## Data Availability

The raw data supporting the conclusions of this article will be made available by the authors on request.

## Supplementary Materials

The following supporting information can be downloaded at: https://www.mdpi.com/article/10.3390/ani15233475/s1, Figure S1: Comparison of AOB between females and males; Figure S2: Comparison of size and histological components of VNO between females and males; Figure S3: Comparison of histological features of sensory epithelium and vomeronasal glands between females and males; Figure S4: Lectin binding in sensory epithelium of a female; Figure S5: Lectin binding in non-sensory epithelium of a female.
