# Supplementary material for: Histochemical Properties of the Vomeronasal System in Hokkaido Sika Deer (Cervus nippon yesoensis)
Source: Animals (Basel). 2025 Dec 2;15(23):3475. doi: 10.3390/ani15233475 (PMC12691102; doi:10.3390/ani15233475)
Supplement: Supplementary file 1 [file animals-15-03475-s001.zip › animals-3989613-supplementary.pdf]

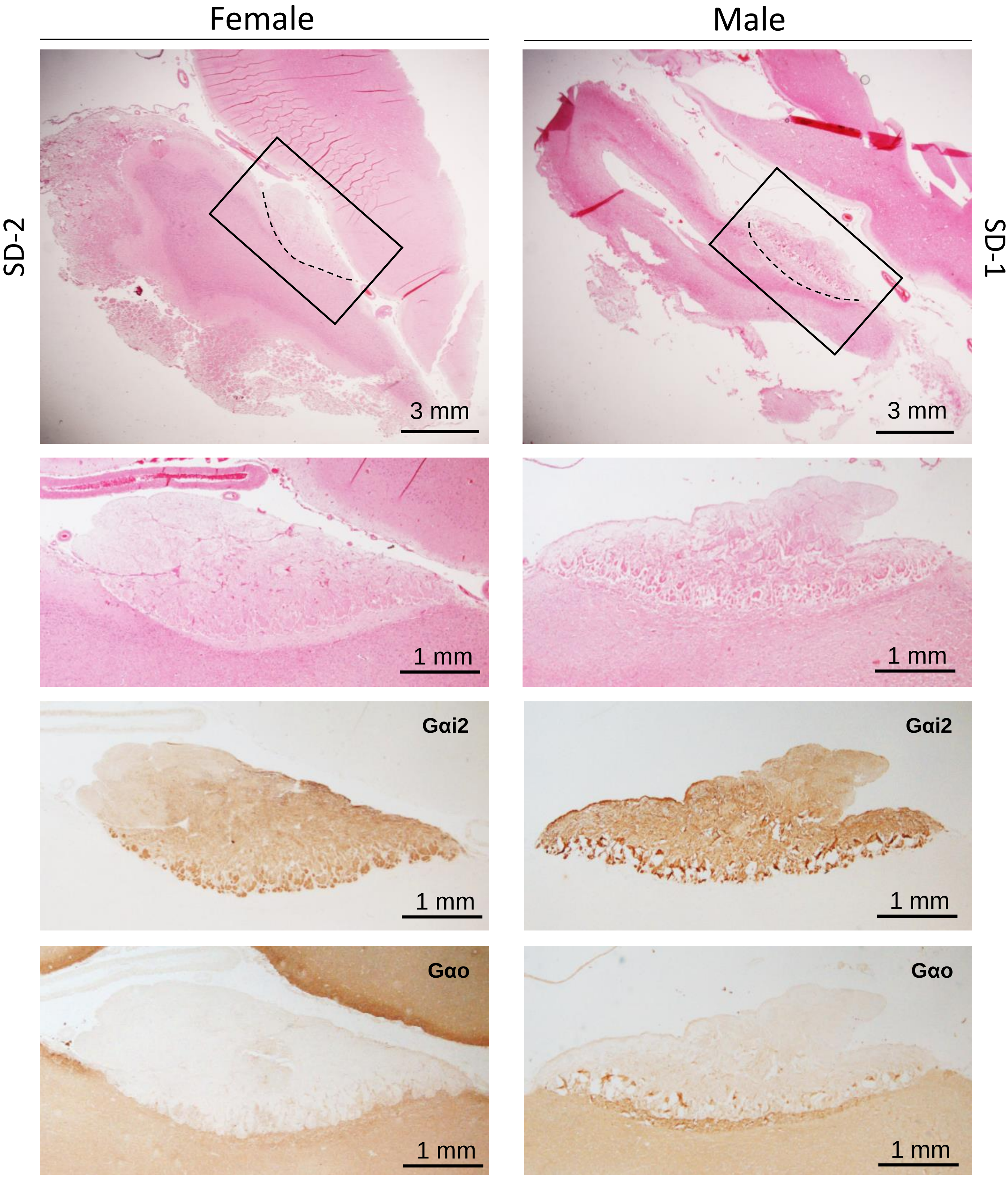

Supplementary Figure S1. Comparison of AOB between female and male sika deer. There is no significant sex difference in this study.

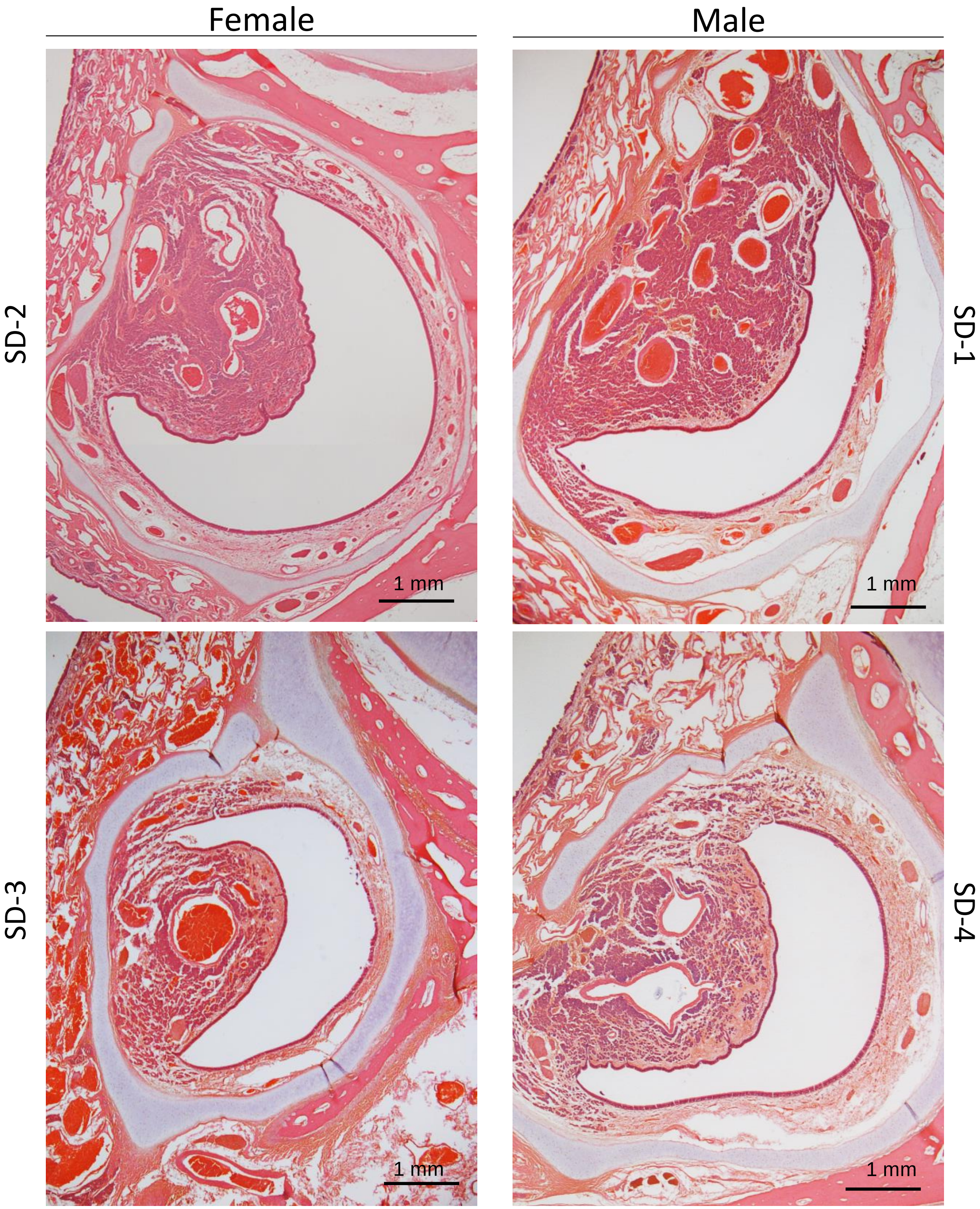

Supplementary Figure S2. Comparison of size and histological components of VNO between female and male sika deer. There is no significant sex difference in this study.

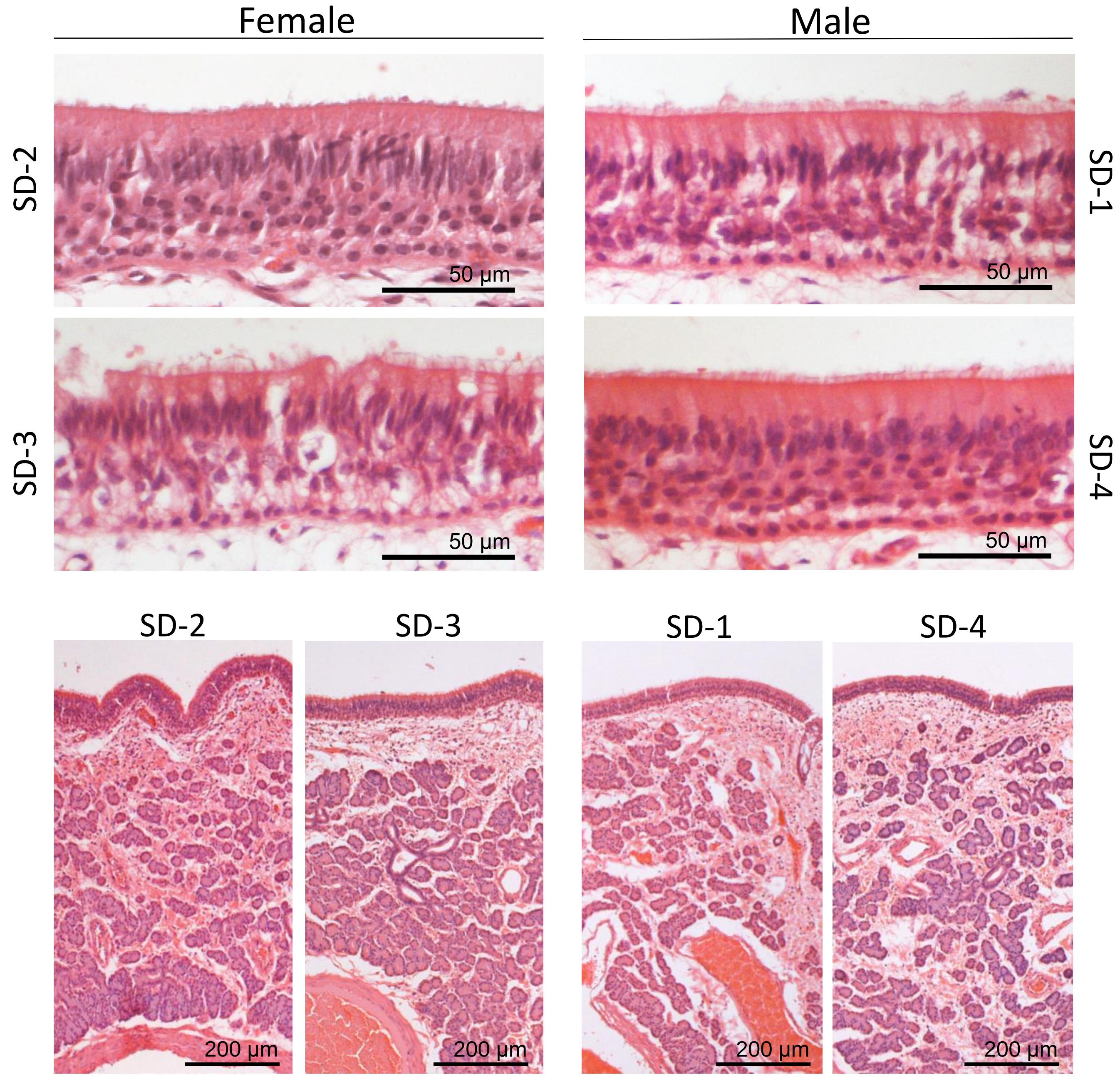

Supplementary Figure S3. Comparison of histological features of sensory epithelium of VNO (upper) and vomeronasal glands (lower) between female and male sika deer. There is no significant sex difference in this study.

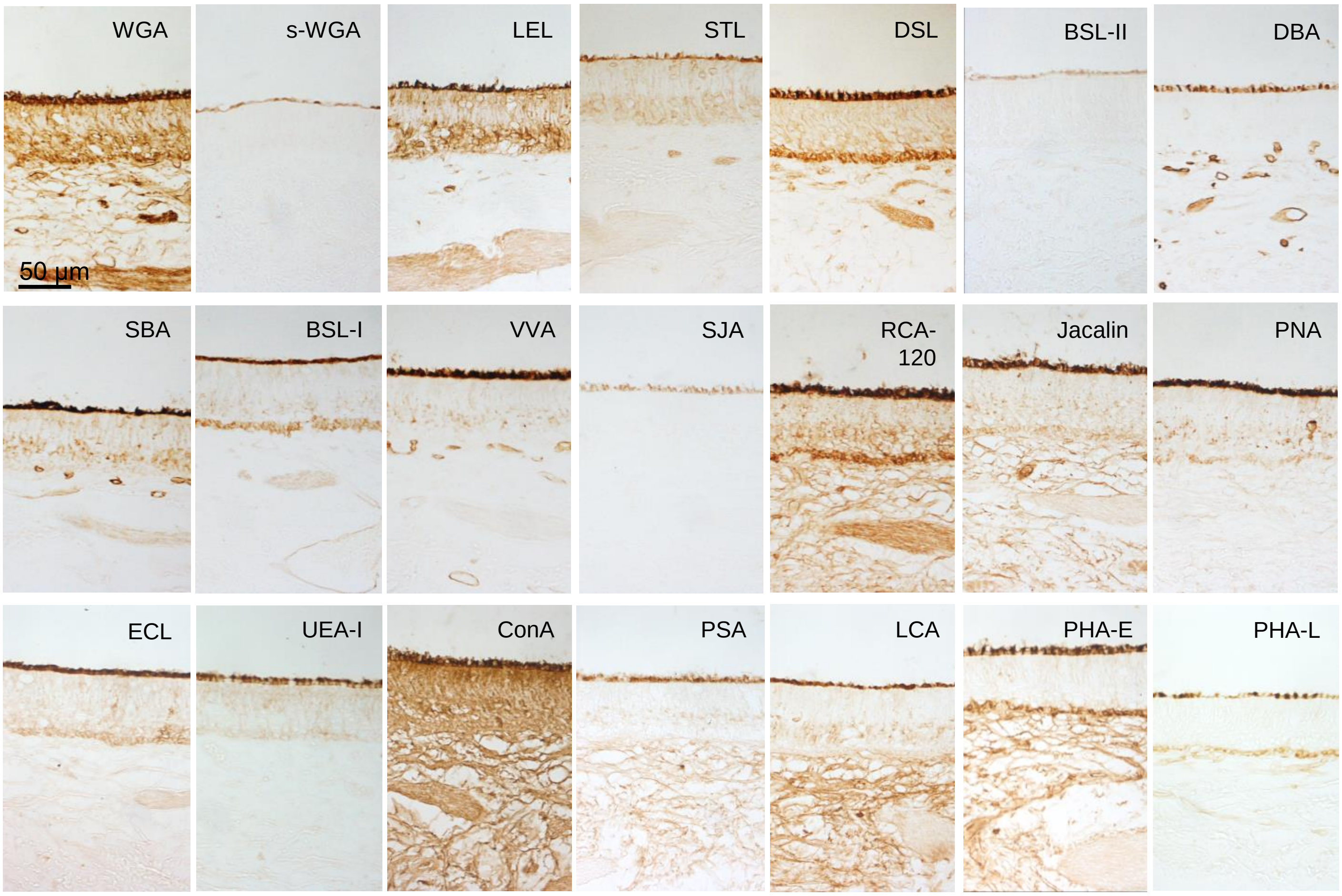

Supplementary Figure S4. Lectin binding in sensory epithelium of a female SD-3. There is no significant difference between females and males (Figure 6).

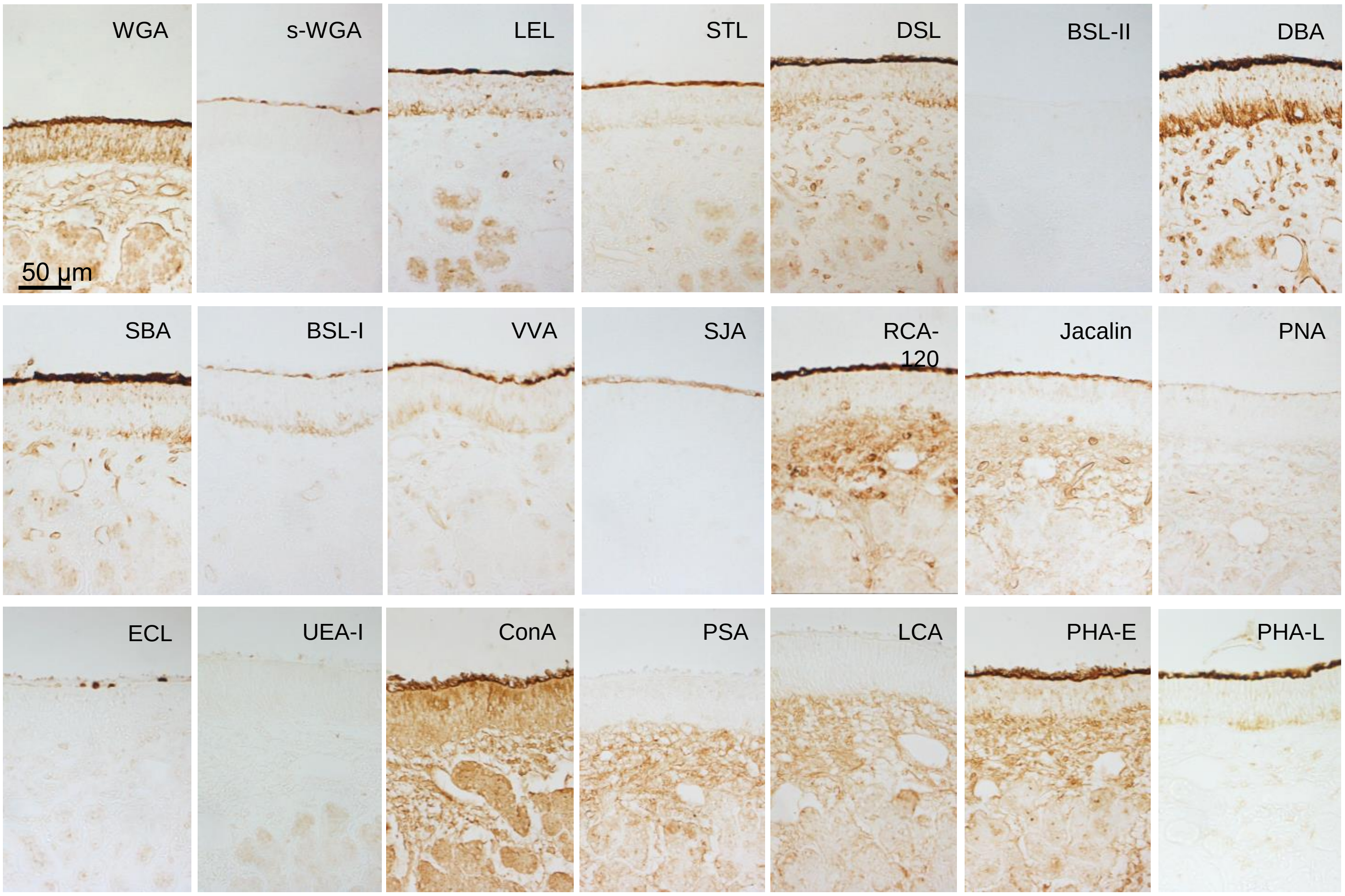

Supplementary Figure S5. Lectin binding in non-sensory epithelium of a female SD-3. There is no significant difference between females and males (Figure 7).
